# Deep brain stimulation for dystonia

**DOI:** 10.1007/s00415-016-8137-9

**Published:** 2016-05-02

**Authors:** Katharine E. Harding, Neil P. Robertson

**Affiliations:** Institute of Psychological Medicine and Clinical Neurosciences, Cardiff University, Cardiff, CF14 4XW UK

The dystonias are a relatively rare group of movement disorders that often prove difficult to treat effectively. In recent years, advances in our understanding of genetics has allowed identification of a number of causative mutations, as well as acquired aetiologies including peri-natal insults. However, despite this progress, pharmacological treatment options have remained limited and patients may still acquire considerable disability as a consequence of the disease. In contrast, the use of deep brain stimulation (DBS), available since the late 1980s, has become more widespread and now has established efficacy in a range of movement disorders, although its exact mode of action remains unclear. A detailed understanding of mechanism of DBS would clearly have significant benefits including refining patient selection and minimising the risk of side effects. Rather uniquely, the procedure itself may offers unparalleled opportunities for direct electrophysiological recordings of brain activity which in turn may allow insights into the pathophysiology underlying movement disorders, and also the mechanism by which it has its effects.

In this month’s journal club, we review three papers exploring DBS in dystonia. The first paper compares detailed electrophysiological recordings in patients with Parkinson’s disease and cervical or limb dystonia, describing patterns of cortical electrical activity and observing the effects of DBS. The second paper uses real-world clinical data to observe globus pallidus neuron firing rates and patterns, correlating these with clinical outcome data in paediatric patients with primary or secondary dystonias 1 year post-operatively. Finally the third paper reports a small series of patients with myoclonus dystonia undergoing DBS, focussing on symptom improvement, quality of life, and need for additional pharmacological treatment at 1 year.

## Patterns of cortical synchronisation in isolated dystonia compared with Parkinson disease

Although DBS is recognised to have an effect in both Parkinson’s disease (PD) and dystonia, the reasons for its effect are not clearly understood. The fact that it is effective in both conditions suggests shared mechanisms. This paper describes electrophysiological findings in the motor cortex in dystonia compared to PD, aiming to improve understanding of underlying pathophysiology. Twenty-two patients with dystonia (8 limb, 14 cervical), and 14 patients with akinetic-rigid PD were studied. Intraoperative recordings were made from the M1 motor strip during rest and also while performing arm movements, prior to implantation of the DBS. In all three groups, increased phase-amplitude coupling was observed, least noticeably in the cervical dystonia group. The authors report that these data suggest oversynchronisation of the motor cortex, which results in impaired initiation and execution of movement. Furthermore, DBS reduced phase-amplitude coupling in a subgroup of four dystonia patients studied both before and during the procedure.

*Comment.* This study has shed some useful light on the electrophysiological mechanisms underlying dystonia, and the extent to which the cortex is involved. The findings suggest that the therapeutic effect of DBS is to reduce over synchronisation of the motor cortex, allowing effective movement. It would certainly be of value to see these findings replicated in a larger dataset and to explore whether similar findings are observed in tremor-dominant PD, or whether the electrophysiological findings in the cortex of such patients are different. This would clearly have important implications on patient selection in maximising chances of response to DBS.

Miocinovic S et al (2015) JAMA Neurol 72(11):1244–1251.

## Differences in globus pallidus neuronal firing rates and patterns relate to different disease biology in children with dystonia

This study uses data collected during routine clinical practice to analyse patterns of firing from globus pallidus neurons and association of these patterns with outcomes at 1 year following DBS. Children undergoing DBS for dystonia were included, and classified as either primary dystonia (with normal imaging) or secondary dystonia. Secondary dystonia was further subdivided into static lesions (such as extreme prematurity, vascular event, or hypoxic ischaemic encephalopathy), and progressive dystonias associated with neurodegenerative conditions such as PANK-2 deficiency. Data were recorded from the globus pallidus interna (GPi) and globus pallidus externa (GPe), and included rate and pattern of firing (burst, regular, or irregular). Clinical outcomes were defined as improvement in the movement scale of the Burke–Fahn–Marsden Dystonia Rating Scale (BFMDRS-m).

Firing rates were higher in both GPi and GPe in primary dystonia and secondary progressive groups compared to secondary static. For primary and secondary static groups, most GPi cell firing was non-regular, but in the secondary progressive group most GPi cells fired in a regular pattern. There was no difference in firing patterns between groups for GPe cells.

At baseline, a trend was noted towards association of lower GPi firing rates with more severe dystonia. At 1 year, there was significant association between higher GPi firing rates and percentage improvement in the BFMDRS-m score. When each group was analysed separately, this effect was most marked in the secondary static group.

*Comment*. A strengths of this paper is in its use of real-world clinical data to analyse electrophysiological findings and outcome in DBS. The observation of an association between higher baseline firing rates and outcome at 1 year is of value, and may be applicable in clinical practice for counselling patients on expectations for treatment response. Furthermore these data may offer future opportunities in establishing markers of prognosis in children undergoing DBS.

McClelland VM et al (2016) JNNP 2016; E-pub ahead of print: 4 Feb 2016. doi:10.1136/jnnp-2015-311803

## Early deep brain stimulation in patients with myoclonus-dystonia syndrome

Myoclonus dystonia is a rare movement disorder often with onset during childhood. In recent years, causative mutations have been identified in the ε-sarcoglycan (*SGCE*) gene, and there are reports in a small number cases that have responded to DBS. However, efficacy of DBS remains unproven in patients negative for *SGCE* mutations. This study reports outcome in two patients with myoclonus dystonia, one with a proven *SGCE* mutation, and one with probable myoclonus dystonia by clinical criteria but negative for known pathogenic mutations. Standard rating scales were used to score symptoms of myoclonus (Unified Myoclonus Rating Scale, UMRS) and dystonia (BFMDRS) pre-operatively and at 6 and 12 months, and the SF-36 was used to measure quality of life at baseline and 12 months. The authors also take the opportunity to summarise previous clinical reports of DBS in myoclonus dystonia.

At 12 months, there was clear improvement in myoclonus for both patients of approximately 70 % and for dystonia of 37 and 86 %. SF-36 scores improved or stabilised. One patient was able to come off medication altogether, while the other was able to stop one medication and substantially reduce the dose of a second.

*Comment.* The limited literature currently available suggests that DBS is of value for symptom control in myoclonus dystonia, although may be more effective for symptoms of myoclonus than dystonia. This case series supports these observations, and suggests it may also be of value in gene-negative patients. However, it is important to note that the aetiology of gene-negative myoclonus dystonia remains unclear, and is likely to differ from disease due to *SGCE* mutations, which could have important implications for treatment. One of the most useful parts of this paper was the clear summary of previous studies on DBS in myoclonus dystonia, suggesting that DBS is effective particularly in patients known to have a mutation in the *SGCE* gene. The next step would clearly be a multi-centre collaboration to investigate long-term outcome in this rare group of patients.

Rocha H et al (2016) J Clin Neurosci 27:17–21.

